# How will Somali coastal upwelling evolve under future warming scenarios?

**DOI:** 10.1038/srep30137

**Published:** 2016-07-21

**Authors:** M. deCastro, M. C. Sousa, F. Santos, J. M. Dias, M. Gómez-Gesteira

**Affiliations:** 1 EPHYSLAB, Environmental PHYsics LABoratory, Facultad de Ciencias, Universidad de Vigo, 32004 Ourense, Spain; 2 CESAM, Departamento de Física, Universidade de Aveiro, 3810-193 Aveiro, Portugal

## Abstract

Somali upwelling system, the fifth in the world, presents some unique features compared with the other major upwelling systems: 1) it is a Western Boundary Upwelling System located near the Equator and 2) upwelling affects the moisture responsible for monsoon rainfall. The intensity of Somali coastal upwelling during summer was projected for the twenty first century by means of an ensemble of Global Climate Models and Regional Climate Models within the framework of CMIP5 and CORDEX projects, respectively. Regardless global or regional circulation models and the chosen greenhouse warming scenario, the strengthening of Somali coastal upwelling, which increases with latitude, is even higher than observed for the Eastern Boundary Upwelling System. In addition, coastal upwelling strengthening is mainly due to Ekman transport since Ekman pumping shows no clear trend for most of the latitudes. Projected land-sea air temperature and pressure show a clear intensification of land-sea thermal and pressure gradient as a consequence of the global warming, which is likely to affect the strengthening of Somali upwelling verifying the hypothesis of Bakun. As a consequence, projected sea surface temperature warming is less intense nearshore than at oceanic locations, especially at latitudes where upwelling strengthening is more intense.

The ecological and socio-economic impact of coastal upwelling along Eastern Boundary Upwelling Systems (EBUS) has been extensively documented in the past, mainly related to the productivity of fisheries^1^ or to the distribution of marine biodiversity^2^. In 1990 Bakun^3^ hypothesized the strengthening of upwelling intensity along the major upwelling ecosystems due to the increase in ocean-land thermal gradient induced by global warming. Since the hypothesis of Bakun, different studies^4^,^5^,^6^,^7^,^8^,^9^ dealing with coastal upwelling intensification show contradictory results highly dependent on the area, the season and the database. In this sense, wind intensification has been analyzed within the framework of global warming for the four major EBUS^4^: Benguela, California, Humboldt and Canary. Sydeman *et al*.^4^ shows that the first three upwelling ecosystems have suffered wind intensification, which was found stronger at higher latitudes consistently with the warming pattern associated to climate change. Other authors^5^ also found upwelling strengthening in coastal areas of Benguela, Peru, Canary and northern California using reanalysis data over the period 1982–2010. These trends were significant only in the last two systems. In contrast, they found significant upwelling weakening along Chile, southern and central California coasts.

Somalia can be considered the fifth most important upwelling system^10^ worldwide in terms of the volume of wind induced upwelled water. Nevertheless, one of the main differences between this system and the rest is the rapid offshore export of surface water^11^ which results in a short residence time inside the nearshore area^12^,^13^. This is due to the presence of the Great Whirl^14^,^15^ in the vicinity of the upwelling zone. In Somalia, coastal upwelling reduces considerably the sea temperature of the Indian Ocean during the summer monsoon (see refs 16 and 17 and the references there in). The Indian warm pool, which is the warmest in the world in spring, represents the most important moisture source for monsoon rainfall^18^. This rainfall is essential for densely populated areas like India since changes in its strength can result in serious damages^19^. Studies focused on upwelling trends along the Somali upwelling system are controversial and depend on the database and the period under study. Within this context, upwelling enhancement has been observed from 1997 to 2004 using NCEP-NCAR reanalysis data^20^, while upwelling decrease has been described from 1979 to 2006 using the same database^16^, as well as over the period 1982–2010^5^ using reanalysis data. In addition, a decrease in the zonal wind component has also been detected over the period 1950–2010^21^.

According to the previously cited scientific literature (see for example refs 4, 5 and 22), it is clear that historical upwelling trends have been properly characterized along the major coastal upwelling regions worldwide. Thus, the question to be answered now is how these coastal upwelling systems will evolve under different greenhouse warming scenarios along the twenty first century. This question was partially addressed by means of an ensemble of climate models developed for the Coupled Model Intercomparison Project phase 5 (CMIP5) along the four major EBUSs^23^. Three of these EBUS (Canary, Benguela and Humboldt) showed a significant increase for projected upwelling, especially at high latitudes. In contrast, an uncertain pattern was observed along the California system. An interesting analysis on the implications of these changes is provided by Bakun *et al*.^9^ and Di Lorenzo^24^.

The objective of this study is to analyze how the Somali coastal upwelling will evolve during the twenty first century. This evolution was investigated by means of global and regional circulation models (GCMs and RCMs) under two different greenhouse warming scenarios. Causes and implications of coastal upwelling evolution along the twenty first century were evaluated assessing the projections of land-sea thermal difference, cross-shore pressure gradient and sea surface temperature from 2006 to 2099.

## Data and Methodology

Near surface (10 m) monthly zonal and meridional wind components from 2006 to 2099 were obtained from seven simulations carried out with GCMs developed for the CMIP5 project (http://cmip-pcmdi.llnl.gov/cmip5/) and from fourteen simulations carried out with RCMs obtained from CORDEX project (http://www.cordex.org/). These regional simulations were produced using four RCMs forced by the seven different GCMs (Table 1). Table 1 summarizes the main characteristics of GCMs and RCMs used in this study. The spatial resolution of RCMs (~50 km) is considerably finer than provided by GCMs, which is typically on the order of 100–210 km (Table 1).

Air temperature, surface air pressure and sea surface temperature (SST) data were also obtained from the seven simulations carried out with GCMs. Because GCMs have different horizontal resolutions (Table 1), these variables were bilinearly interpolated on a common 1° × 1° grid.

The study was carried out simulating different future warming scenarios (Representative Concentration Pathways, RCPs from now on). Two greenhouse warming scenarios were considered: RCP 8.5 and RCP 4.5 which exhibit a radiative forcing of 8.5 and 4.5 Wm^−^^2^, respectively, at the end of twenty first century^25^.

Offshore wind-driven Ekman transport was used to calculate the upwelling index (UI) following the procedure described in Santos *et al*.^15^. Ekman pumping (EP) was calculated from wind components following the procedure described in Alvarez *et al*.^26^.

**Figure 1:**
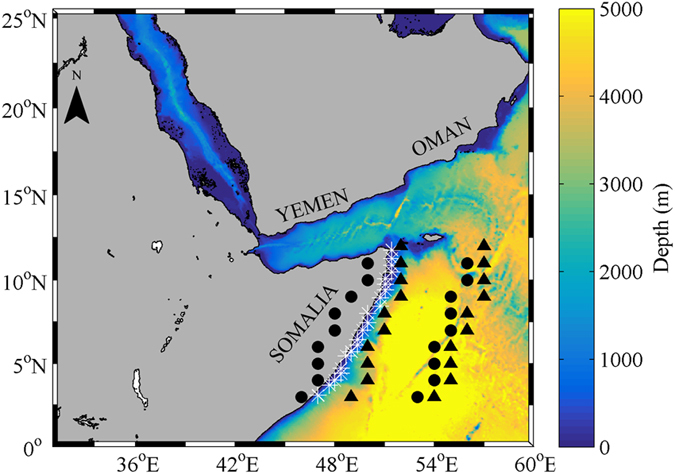
Bathymetry (m) of the area under scope. White asterisks represent the 21 points at approximately 50 km from coast to calculate coastal upwelling trends from RCMs. Black points represent points at 0.5° inland from coastline and 5° offshore to calculate trends of: land air temperature, land-sea temperature difference and land-sea air pressure difference, using GCMs. Black triangles represent points at 1° and 6° offshore to calculate trends of offshore-coast SST differences from GCMs. Points 1° offshore were also used to calculate trends of: coastal upwelling, coastal SST and EP, using GCMs. This figure was produced using MATLAB version R2015b (http://www.mathworks.com).

Upwelling, air temperature, surface air pressure and SST trends were calculated during the summer monsoon (June-August): the period of the year characterized by strong SW winds blowing along the Somali coast^15^. 21 points distributed along the Somali coast at approximately 50 km from coast were considered to calculate coastal upwelling trends from RCMs (Fig. 1, white asterisks). Points at approximately 0.5° inland from coastline and 5° offshore were considered to calculate trends of: land air temperature, land-sea temperature difference and land-sea air pressure difference from GCMs (Fig. 1, black points). Points at approximately 1° and 6° offshore were considered to calculate trends of offshore-coast SST difference (Fig. 1, black triangles). Points 1° offshore were also considered to calculate trends of: coastal upwelling, coastal SST and EP, from GCMs.

A multimodel mean was considered to simplify results due to its lower biases, errors and uncertainties compared with each individual model^27^,^28^,^29^. In addition, the standard deviation divided by the square root of the number of models was also calculated. Following the procedure described in Tebaldi *et al*.^30^ and Wang *et al*.^23^, values are considered statistical robust when at least 50% of the climate models show a significant trend (p < 0.05) and at least 80% of those have the same trend sign.

## Results and Discussion

**Figure 2:**
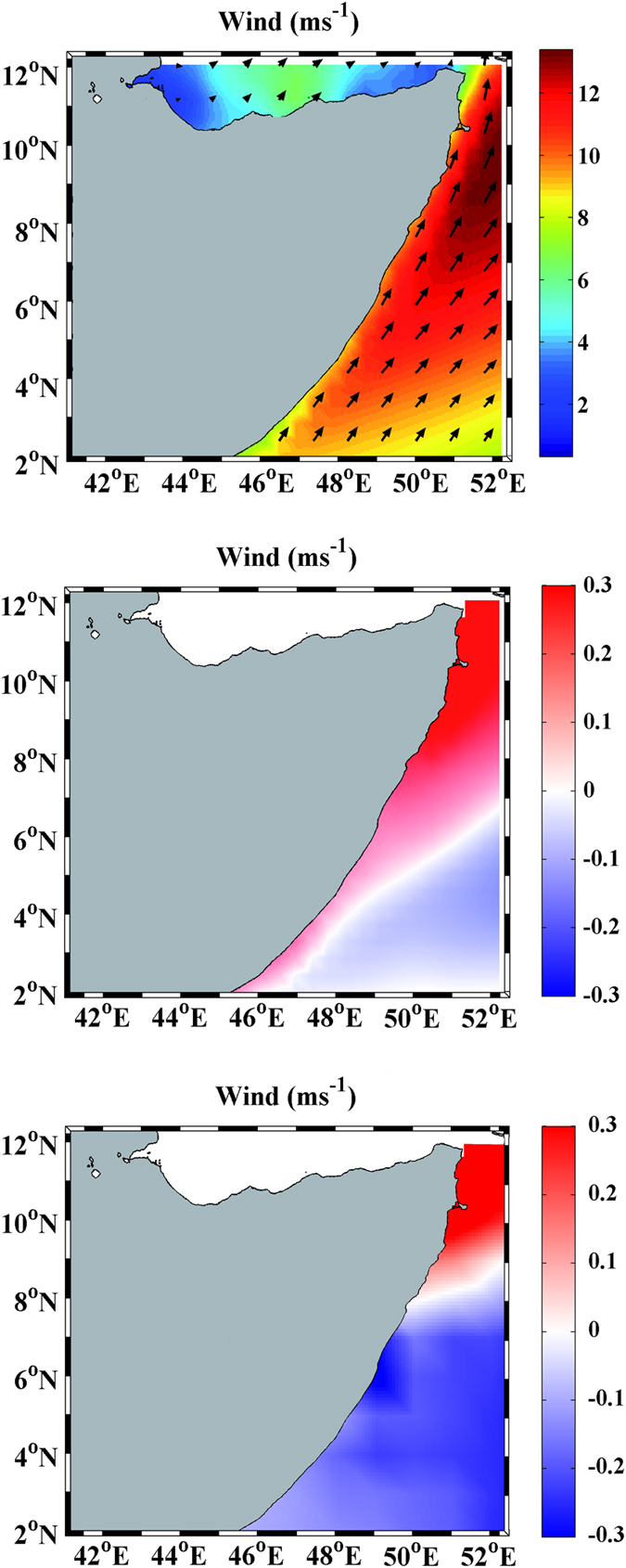
**(a)**Present wind field (ms^−1^) from RCMs along the Somali coast averaged during summer monsoon (June–August) from 2006 to 2015. Colour represents the module of wind speed. Difference of alongshore component of summer wind speed (ms^−1^) calculated during 2090s (future) and averaged from 2006 to 2015 (present) from RCMs **(b)** and GCMs **(c)**. All figures are a multimodel mean calculated under RCP 8.5 scenario. This figure was produced using MATLAB version R2015b (http://www.mathworks.com).

Summer wind field averaged from 2006 to 2015 is characterized by SW winds blowing along the Somali coast (Fig. 2a). This wind field pattern is a multimodel mean using RCMs under RCP8.5 scenario. Wind intensity higher than 12 ms^−1^ was observed in the northern part of Somalia. These intense SW winds generate a strong coastal upwelling off Somalia, which is consistent with the pattern described by Santos *et al*.^3^. Differences in the alongshore component of summer wind speed calculated during 2090s (future) and averaged from 2006 to 2015 (present) show a coastal wind increment along the whole Somali coast with higher values, 0.3 ms^−1^, at northern latitudes (Fig. 2b). This coastal wind increment is only visible at northern latitudes when GCMs were considered to calculate alongshore winds (Fig. 2c) due to their coarse resolution, which becomes more patent nearshore.

**Figure 3:**
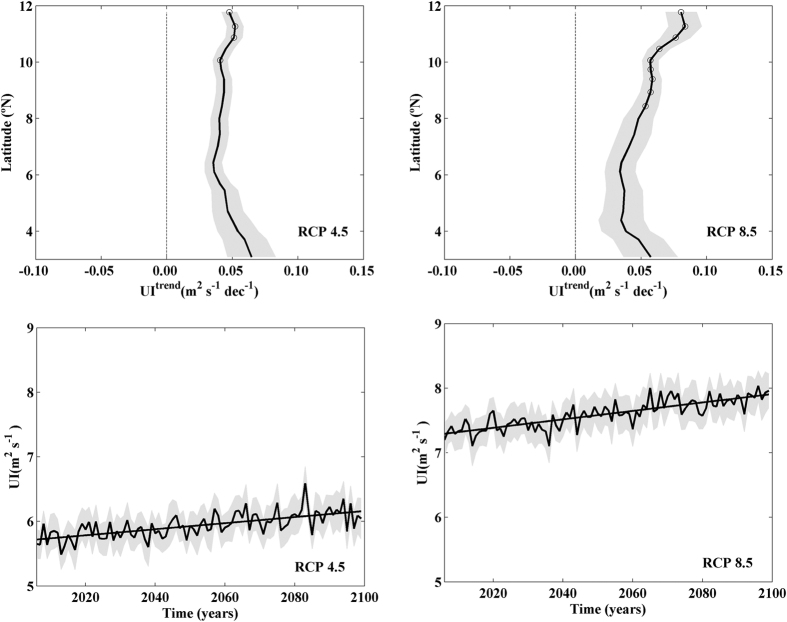
UI linear trends (m^2 ^s^−1^ dec^−1^) for the 21 points depicted in Fig. 1 projected during summer (June– August) from 2006–2099 using RCMs under RCP 4.5 (**a)** and 8.5 **(b**) scenarios. Interannual evolution of UI (m^2 ^s^−1^) under RCP 4.5 (**c**) and 8.5 (**d**) scenarios. The UI signal was spatially averaged for those latitudes with statistical robust values of UI trends. Multimodel mean (solid line) and standard deviation (shading). Circles represent trends that are robust across climate models. This figure was produced using MATLAB.

Summer upwelling trends were calculated along the Somali coast projecting UI by means of RCMs under RCP 4.5 and 8.5 scenarios over the period 2006–2099 (Fig. 3a,b). The dark line represents the multimodel mean and the grey shadow its standard deviation divided by the square root of the number of models. Circles represent statistically robust values. Upwelling increase was observed for all Somali latitudes under both scenarios reaching statistically robust values (higher than 0.05 m^2^ s^−1^ dec^−1^) for latitudes north of 8.5 °N. The increasing pattern is more marked under the RCP 8.5 scenario (Fig. 3b) where statistically robust values ranging from 0.05 to 0.08 m^2^ s^−1^ dec^−1^ can be observed north of 8°N. The increase observed at high latitudes over the whole period is close to 10% of the mean upwelling intensity. The strengthening in Somali coastal upwelling, which increases with latitude, is even higher than observed for Humbolt, Benguela and Canary coastal upwelling systems^23^, where strengthening ranges from 0 to 0.01 m^2^ s^−1^ dec^−1^. However, it should also be taken into account that the mean upwelling intensity is also higher for Somalia due to its proximity to the Equator. The inter-annual evolution of summer UI projected under both scenarios is averaged for those latitudes which showed statistically robust values for UI trends (Fig. 3c,d). A significant increasing trend in coastal upwelling is observed under both scenarios. This increase is on the order of 0.05 m^2^ s^−1^ dec^−1^ under the RCP 4.5 (Fig. 3c) and 0.07 m^2^ s^−1^ dec^−1^ under the RCP 8.5 (Fig. 3d).

Both greenhouse warming scenarios show similar upwelling strengthening patterns, however less intense in the RCP 4.5. From now on only the most unfavourable scenario will be considered (RCP 8.5) for the sake of clarity.

**Figure 4:**
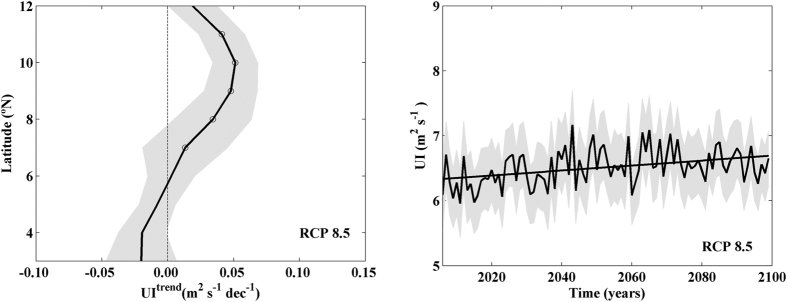
(**a)** UI linear trends (m^2 ^s^−1^ dec^−1^) calculated for summer (June– August) from 2006–2099 using GCMs under RCP 8.5 scenario. Trends were calculated considering points depicted in Fig. 1. (**b**) Interannual evolution of UI (m^2^s^−1^) under the RCP 8.5. The UI signal was spatially averaged for those latitudes with statistical robust values of UI trends. Multimodel mean (solid line) and standard deviation (shading). This figure was produced using MATLAB.

Summer upwelling pattern along the Somali coast projected for the twenty first century using GCMs share most of features previously observed using RCMs (Fig. 4a). Upwelling increases for latitudes north of 6°N with significantly robust values close to 0.05 m^2^ s^−1^ dec^−1^ for latitudes between 8°N and 11°N. Practically null values without significance were observed south of 6°N. In addition, a significant strengthening in coastal upwelling is obtained when UI is averaged for latitudes with significant upwelling trends (Fig. 4b). Coastal upwelling strengthening from GCMs (0.04 m^2^ s^−1^ dec^−1^) is weaker than the one obtained from RCMs (0.07 m^2^ s^−1^ dec^−1^) under the RCP 8.5 scenario.

**Figure 5:**
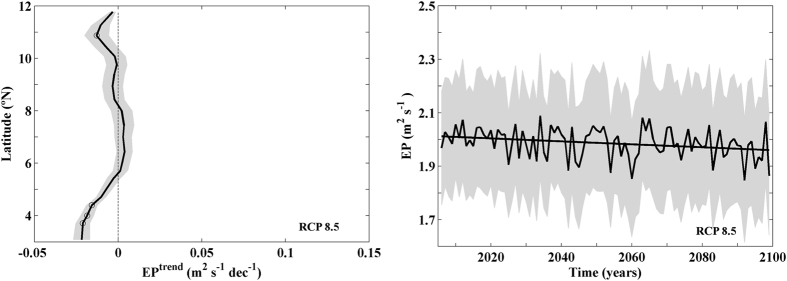
**(a)**Ekman pumping (EP) linear trends (m^2^ s^−1^ dec^−1^) for the points depicted in Fig. 1 projected during summer (June–August) from 2006–2099 using RCMs under RCP 8.5 scenario. (**b**) Ekman pumping (EP) interannual evolution (m^2 ^s^−1^). EP signal was spatially averaged. Multimodel mean (solid line) and standard deviation (shading). This figure was produced using MATLAB.

Another mechanism that can be responsible of changes in the amount of water advected to surface is Ekman pumping. This mechanism, which depends on wind curl, is at least four times smaller than Ekman transport which depends on wind strength. Thus, more than 80% of the upwelled water in this area is due to Ekman transport. Summer EP trends along the Somali coast over the twenty first century show a decreasing pattern with values close to zero (without significance) for latitudes north of 5°N (Fig. 5a). Statistically robust EP trends were only obtained at those latitudes where the projected Ekman transport shows no significant trends. The interannual evolution of the spatially averaged EP shows a decreasing trend of 0.0055 m^2^ s^−1^ dec^−1^ which represents a decrement on the order of 2.5% of the mean EP intensity over the whole period of time (Fig. 5b). Ekman pumping trends were considerably less intense than previously shown for upwelling index due to Ekman transport (Fig. 3). Finally, changes in the actual amount of water pumped to the surface cannot only be due to Ekman transport and pumping since changes in the stratification can also play an important role by enhancing or reducing the ability of water to respond to wind forcing.

**Figure 6:**
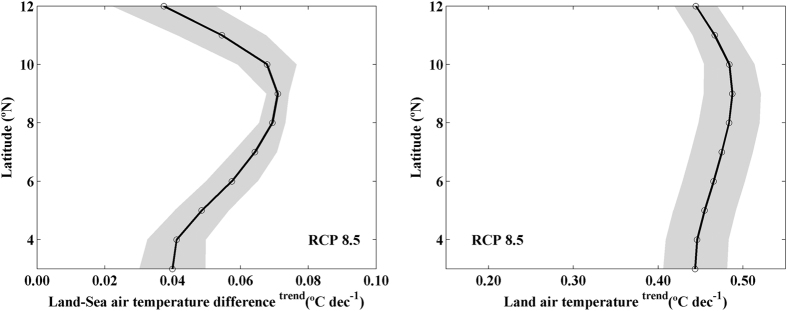
**(a)**Linear trends of land-sea air temperature difference and **(b)** Air temperature linear trends (°C dec^−1^) during summer monsoon (June–August) from 2006 to 2099 using GCMs under RCP 8.5 scenario. Trends were calculated considering the points depicted in Fig. 1. Multimodel mean (solid line) and standard deviation (shading). Circles represent trends that are robust across climate models. This figure was produced using MATLAB.

**Figure 7:**
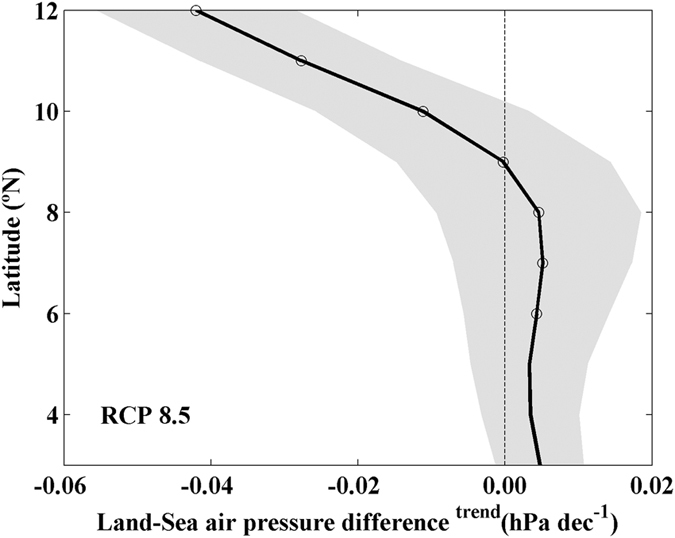
Linear trends of land-sea air pressure difference (hPa dec^−1^) during summer monsoon (June–August) from 2006 to 2099 using GCMs under RCP 8.5 scenario. Trends were calculated considering the points depicted in Fig. 1. Multimodel mean (solid line) and standard deviation (shading). Circles represent trends that are robust across climate models. This figure was produced using MATLAB.

There are several factors that make Somalia a unique upwelling system. First of all, the relative position between ocean and coast since Somalia is a Western Boundary Upwelling System (WBUS) whilst California, Canary, Benguela and Humboldt are EBUS. Second, Somalia is located near the equator whilst the rest of the systems are mainly located at the subtropics. Nevertheless, the Bakun hypothesis^3^ that was invoked in recent publications as responsible for upwelling strengthening^4^,^5^,^9^,^23^,^24^ should remain valid no matter the hemisphere, the relative position between ocean and coast or the proximity to the Equator. In this sense, trends of the projected land-sea thermal difference along the Somali coast were calculated using air temperature from GCMs (Fig. 6a). The strengthening of the land-sea thermal gradient is significant at all latitudes with values ranging from 0.04 to 0.07 °C dec^−1^. This intensification, as a consequence of a global warming (~0.45 °C dec^−1^, Fig. 6b), causes the significant strengthening of the land-sea air pressure difference with values between −0.01 and −0.04 hPa dec^−1^ north of 9°N (Fig. 7). Land-sea air pressure difference is practically negligible south of 9°N. The strengthening of both land-sea thermal and cross-shore pressure gradients are directly correlated with the coastal upwelling strengthening referred above.

Some authors have recently suggested possible changes in the pressure systems (and hence in wind systems) due to future alterations in Hadley Cells^9^,^31^,^32^,^33^ as a possible cause of coastal upwelling strengthening. This can explain how the increase described for Canarias, Humboldt and Benguela^9^ is more intense at higher latitudes and practically negligible at intermediate latitudes. Nevertheless, in the particular case of Somalia, upwelling strengthening is also clear despite the system is located at very low latitudes. It should be noted that the analysis in terms of pressure cells is not straightforward for the North Indian Ocean where the *hypothetical* Hadley cell is disrupted by the presence of land masses. Nevertheless, changes in the seasonal migration of the Intertropical Convergence Zone (ITCZ) can play a key role to understand changes in wind intensity during the upwelling season^15^. Recently Sandeep and Ajayamohan^34^ reported a poleward shift in the Indian summer monsoon. Under a scenario of global warming the land surface has experienced a faster rate of heating compared to the adjacent ocean. This faster heating generates a deepening of the low pressure zone over land which results in the strengthening of the cross- equatorial pressure gradient and, subsequently, in the increase of upwelling favourable winds.

**Figure 8:**
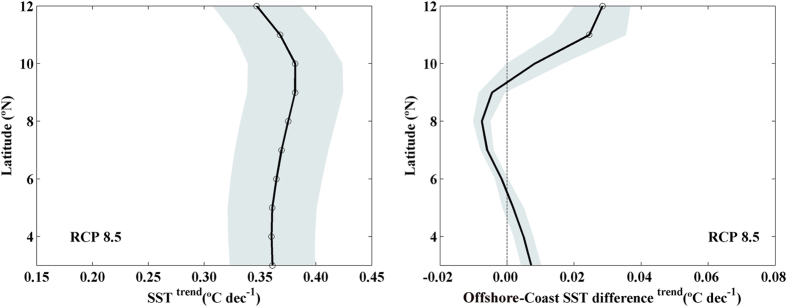
(**a**) SST linear trends and **(b)** Linear trends of offshore-coast SST difference (°C dec^−1^) during summer monsoon (June–August) from 2006 to 2099 using GCMs under RCP 8.5 scenario. Trends were calculated considering the triangles depicted in Fig. 1. Multimodel mean (solid line) and standard deviation (shading). Circles represent trends that are robust across climate models. This figure was produced using MATLAB.

The implications of upwelling strengthening have been widely described for the major EBUS^9^,^24^. Most of the information provided by those authors should also be suitable for the Somali system. In addition, there are some particular features that are specific of the northern part of the Indian Ocean. SST is predicted to decrease under a scenario of upwelling increase. This colder water, which would be rapidly advected to the open ocean due to the presence of the Great Whirl, would affect the rainfall associated to summer monsoon. Because its importance, summer SST trends were evaluated along the Somali coast by means of GCMs projections for the twenty first century (Fig. 8a). A significant coastal SST warming, ~0.35 °C dec^−1^, was obtained along the whole coast being less intense at the northernmost latitudes. A priori, upwelling increase should result in coastal water cooling. Nevertheless, it should be kept in mind the context of global warming projected during the twenty first century which would result in ocean warming worldwide. The strengthening of coastal upwelling can locally alleviate this warming pattern, which would be less intense nearshore. This is in agreement with previous studies carried out in other EBUS^35^,^36^ over the twenty century (northern limit of the Canary upwelling ecosystem and Benguela). These studies prove that SST warming in coastal areas affected by upwelling strengthening has been less intense than at the adjacent ocean. This behavior was also observed along the Somali coast (Fig. 8b) where the difference between offshore and coastal SST trends is significantly positive at the northernmost latitudes where upwelling is projected to strengthen faster (Fig. 3b). Thus, ocean water will warm at a higher rate than coastal water. The rest of latitudes show SST trends close to zero without robust significant values.

In addition to the above mentioned, the stratification of the water column will increase in a context of ocean warming which can counteract the effect of alongshore wind intensification resulting in less effective coastal upwelling. According to previous research^37^, air-atmosphere coupling, on short temporal and horizontal scales (until 20 km), can affect wind stress in upwelling areas playing a key role in controlling essential features of inner-shelf circulation. Thus, the atmospheric boundary layer structure can be altered by air-sea fluxes over the cold upwelled water. SST cooling could result in a reduction of the surface wind stress. Unfortunately, GCMs and RCMs spatial resolutions are too coarse (more than 1° and 0.44° respectively) to analyze this effect.

## Conclusions

Future climate projections were carried out along the Somali upwelling ecosystem in order to analyze the causes and implications of the evolution of summer coastal upwelling along the twenty first century. These projections were carried out by means of GCMs and RCMs under two different greenhouse warming scenarios (RCP 4.5 and 8.5).

All projections provided by GCMs and RCMs show the strengthening of the Somali coastal upwelling with robust values north of 8.5°N. This strengthening is even more marked under the RCP 8.5 scenario. The strengthening of Somali coastal upwelling, which increases with latitude, is even higher than that observed for the major EBUS (Humbolt, Benguela and Canary).

Regardless global or regional circulation models and the greenhouse warming scenario a significant upwelling increase ranging from 0.05 to 0.07 m^2^ s^−1^ dec^−1^ was projected for the whole Somali upwelling ecosystem along the twenty first century.

Projected EP shows no clear trends for most of the latitudes. A significant decrease in the EP is only obtained for the southernmost latitudes. Thus, changes in coastal upwelling projected for the twenty first century are mainly due to the Ekman transport.

Differences in the spatial resolution between GCMs (100–210 km) and RCMs (~50 km) and the number of simulations available in the area under study for the statistical analysis (7 for CMIP5 GCMs *vs* 14 for CORDEX RCMs) make RCMs more appropriate to analyze the significant spatial variability of upwelling intensity in a single upwelling ecosystem.

Projected land-sea air temperature and air pressure differences along the twenty first century show a clear intensification as a consequence of the global warming. This intensification has a strong influence on coastal upwelling strengthening verifying the hypothesis of Bakun.

The most direct implication of a coastal upwelling strengthening is a projected nearshore SST warming less intense than at the adjacent ocean, especially at latitudes where changes in upwelling are projected to be more intense.

Characterization and analysis of future upwelling changes along the Somali upwelling system is of great relevance due to their effect on the productivity and the marine biodiversity of the ecosystem and also to determine modifications in the rainfall regime. Changes in precipitation in densely populated areas like India, with potential dramatic economic and social impact, are of vital economic importance since values above the average may result in flooding and below the average in seasonal drought.

## Additional Information

**How to cite this article**: deCastro, M. *et al*. How will Somali coastal upwelling evolve under future warming scenarios? *Sci. Rep.* **6**, 30137; doi: 10.1038/srep30137 (2016).

## Figures and Tables

**Table 1:** Summary of simulations used in this study.

RCM		CCLM4-8-17	RCA4	RACMO22T	HIRHAM5
GCM		Bettems *et al*.^45^	Jones *et al*.^46^	Meijgaard *et al*.^47^	Christensen *et al*.^48^
CNRM-CM5	1.4° × 1.4°	X	X		
	Voldoire *et al*.^38^				
CSIRO-Mk3-6-0	1.9° × 1.9°		X		
	Jeffrey *et al*.^39^				
EC-EARTH	1.12° × 1.125°	X	X	X	X
	Hazeleger *et al*.^40^				
HadGEM2-ES	1.25° × 1.875°	X	X	X	
	Collins *et al*.^41^				
IPSL-CM5A-MR	1.25° × 2.5°		X		
	Dufresne *et al*.^42^				
MPI-ESM-LR	1.8° × 1.8°	X	X		
	Jungclaus *et al*.^43^				
NorESM1-M	1.9° × 1.5°		X		
	Bentsen *et al*.^44^				

Global and regional computational models from CMIP5 (http://cmip-pcmdi.llnl.gov/cmip5/) and CORDEX (http://www.cordex.org/) projects. The horizontal resolution corresponds to the atmospheric model. The mean resolution of ocean models is about 1°.
